# POTENTIAL IMPLICATIONS OF THE 2011–2012 DROUGHT IN MEXICO ON OLDER ADULT HEALTH

**DOI:** 10.1093/geroni/igae098.0312

**Published:** 2024-12-31

**Authors:** Joseph Saenz, Jorge Peniche, Laura Tanner, Emma Aguila, Brian Downer, Rebeca Wong

**Affiliations:** Arizona State University, Phoenix, Arizona, United States; University of Southern California, Los Angeles, California, United States; Arizona State University, Phoenix, Arizona, United States; University of Southern California, Los Angeles, California, United States; The University of Texas Medical Branch, Galveston, Texas, United States; The University of Texas Health Sciences Center in San Antonio, San Antonio, Texas, United States

## Abstract

**Background:**

Climate change will increase the frequency and intensity of extreme weather events such as heatwaves, wildfires, hurricanes, and droughts. Few studies have evaluated the health impacts of drought exposure in low- and middle-income countries, which are most vulnerable to effects of climate change. This study evaluates how level of exposure to a major drought that affected Mexico from 2011-2012 related with post-drought health in older adults.

**Methods:**

We use the 2012 Mexican Health and Aging Study (n=8,954 respondents age 50+) linked with municipality-level data from the Drought Monitor of Mexico to identify droughts in MHAS respondents’ community of residence from October 2010 to October 2012. Health domains evaluated included stress/mental health, physical health, and cognitive health. Linear, logistic, and multinomial regressions are used to estimate associations between the longest streak of consecutive months that one’s community was in a drought and health, controlling for sociodemographic characteristics.

**Results:**

Six months of additional community-level drought exposure related with worse mental health and stress (higher depressive symptoms [β=-0.04, p<0.001], poorer self-rated health [OR: 1.11, p<0.01], poorer self-rated finances [OR: 1.09, p<0.05]) worse cognitive health (lower Verbal Learning [β=-0.03, p<0.01] and Verbal Recall [β=-0.04, p<0.01]), and increased likelihood of decreasing weight more than 5 kg during the drought (OR: 1.07, p<0.05).

**Discussion:**

Longer exposure to drought throughout the 2011-2012 drought was associated with worse health across multiple health domains. Future studies should seek to identify factors that may increase vulnerability or facilitate resilience in communities affected by drought.

